# Alternative splicing and expression of human and mouse NFAT genes^☆^

**DOI:** 10.1016/j.ygeno.2008.06.011

**Published:** 2008-11

**Authors:** Hanna Vihma, Priit Pruunsild, Tõnis Timmusk

**Affiliations:** Department of Gene Technology, Tallinn University of Technology, Akadeemia Tee 15, Tallinn 19086, Estonia

**Keywords:** NFAT, Alternative splicing, Ca^2+^-regulated transcription factor, Calcineurin, Neurons, Brain, Nervous system

## Abstract

Four members of the nuclear factor of activated T cells (NFAT) family (NFATC1, NFATC2, NFATC3, and NFATC4) are Ca^2+^-regulated transcription factors that regulate several processes in vertebrates, including the development and function of the immune, cardiovascular, musculoskeletal, and nervous systems. Here we describe the structures and alternative splicing of the human and mouse NFAT genes, including novel splice variants for *NFATC1, NFATC2, NFATC3,* and *NFATC4,* and show the expression of different NFAT mRNAs in various mouse and human tissues and brain regions by RT-PCR. Our results show that alternatively spliced NFAT mRNAs are expressed differentially and could contribute to the diversity of functions of the NFAT proteins. Since NFAT family members are Ca^2+^-regulated and have critical roles in neuronal gene transcription in response to electrical activity, we describe the expression of *NFATC1, NFATC2, NFATC3,* and *NFATC4* mRNAs in the adult mouse brain and in the adult human hippocampus using in situ hybridization and show that all NFAT mRNAs are expressed in the neurons of the mouse brain with specific patterns for each NFAT.

Nuclear factor of activated T cells (NFAT) is a family of transcription factors evolutionarily related to Rel/NF-κB [1]. The family consists of the Ca^2+^-regulated members NFATC1 (NFATc, NFAT2), NFATC2 (NFATp, NFAT1), NFATC3 (NFATx, NFAT4), and NFATC4 (NFAT3) and osmotic tension-regulated NFAT5. The approved human symbols for the NFAT family members are NFATC1, NFATC2, NFATC3, NFATC4 and NFATC5 and the approved mouse symbols are Nfatc1, Nfatc2, Nfatc3, Nfatc4 and Nfatc5. The Ca^2+^-regulated NFAT proteins consist of two conserved domains—a regulatory domain in the N-terminus and a Rel homology domain (RHD) in the C-terminus [2]. The regulatory domain consists of two conserved binding sites for the protein phosphatase calcineurin (CaN) [3,4], an extended serine-rich region, and a nuclear localization signal (NLS) [5]. The Rel homology domain binds DNA and interacts with partner proteins (also referred as NFATn) to transactivate gene transcription. The partner transcription factors include AP-1 (FOS or JUN) [6], GATA4 [7], and MEF2 [8], for example. The N- and C-terminal ends of each NFAT family protein are unique and contain transcription activation domains (TADs) [9].

The NFAT proteins exist in at least two alternative conformations—one in which the NLS is exposed and the nuclear export signal (NES) is masked or vice versa. This is achieved by dephosphorylation or phosphorylation, respectively, of serines in the regulatory domain [10]. NFATs are dephosphorylated by CaN [11] and phosphorylated by various kinases [10] such as casein kinase 1 (CSNK1A1), glycogen synthase kinase 3 (GSK3A or GSK3B), p38 MAP kinase (MAPK14), and JUN N-terminal kinase (MAPK8), [12–16]. Exposure of the NLS leads to rapid import of the NFAT proteins into the nucleus, where they bind to DNA and regulate target gene expression [17], whereas phosphorylation causes rapid relocalization out of the nucleus, terminating NFAT-dependent transcription regulation [18].

NFAT proteins regulate gene transcription in various developing and adult tissues. For example, their roles in the immune system [2], cardiovascular system [2,19], skeletal muscle [20], and nervous system [21,22] have been described. Accordingly, NFAT genes are expressed in almost all tissues. However, the expression levels and patterns for each NFAT are rather distinct [17,23]. All of the NFAT genes except *NFATC4* are strongly expressed in the immune system, in the thymus, spleen, and peripheral blood lymphocytes [23–28], but are also expressed at lower levels in other tissues. *NFATC1* has been detected in the cardiovascular and digestive systems, for example [26,29,30], and *NFATC2* expression has been detected in the testis, pancreas, placenta, and brain–in the hypothalamus, hippocampus, cerebellum, olfactory bulb, and frontal cortex [23,24,31–33]. In addition to the immune system, *NFATC3* is expressed in the skeletal and smooth muscle, kidney, and lung and in the brain, where it has been shown to be expressed in the hypothalamus and striatum [26,28,31,34,35]. *NFATC4* is more evenly expressed than the other NFAT genes and its expression has been detected in the placenta, lung, kidney, adipose tissue, cardiac muscle, testis, ovary, digestive system, and spinal cord and, at lower levels, in the brain—in the hippocampus, cerebellum, olfactory bulb, and various hypothalamic nuclei [19,21,23,34,36–40].

Despite this information, the expression of different NFAT isoforms generated by splicing or usage of alternative 5′ and 3′ exons has not been studied. Therefore, here we describe the structures of the human and mouse *NFATC1, NFATC2, NFATC3,* and *NFATC4* genes and analyze their alternative splicing and coding potentials. Furthermore, we have studied the expression of different *NFATC1, NFATC2, NFATC3,* and *NFATC4* mRNA splice variants in various mouse and human tissues and brain regions by RT-PCR and describe here the expression of the NFAT mRNAs in the adult mouse brain and in the adult human hippocampus using in situ hybridization.

## Results

### The structure of the human and mouse NFAT genes

The exon/intron structures of the human and mouse NFAT genes were characterized and the alternative splicing patterns of each NFAT gene in both human and mouse were analyzed using bioinformatics and RT-PCR. For each NFAT a search for mRNA sequences and expressed sequence tags (ESTs) was performed. RT-PCR analyses were used for the characterization of the expression patterns of the alternative transcripts in human and mouse.

**Fig. 1:**
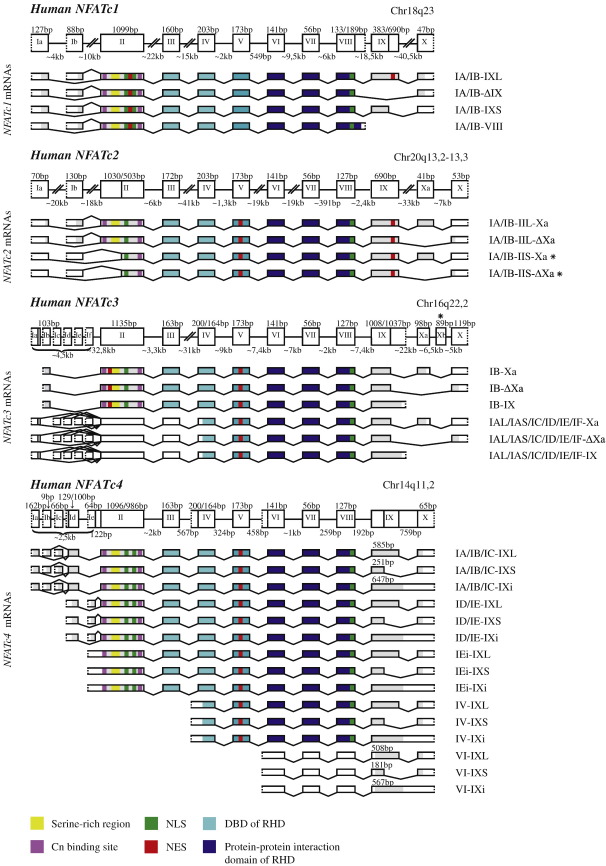
Structure and alternative transcripts of human NFAT genes. The structural organization of human *NFATC1, NFATC2, NFATC3,* and *NFATC4* was determined by analyzing genomic and mRNA sequence data using bioinformatics and RT-PCR. Exons are shown as boxes and introns are shown as lines. The numbers above the exons indicate the size of the protein coding part of the exon. Protein coding sequences of the mRNAs are shown as filled boxes and open boxes indicate UTRs of the mRNAs. Numbers below the introns indicate their size. Exon numbers are shown in roman characters. Asterisks mark rarely used exons and rarely transcribed mRNA variants. NES, nuclear export signal; NLS, nuclear localization signal; DBD, DNA binding domain; RHD, Rel homology domain; Cn, calcineurin A.

The lengths of the four human NFAT genes vary from 10kb for *NFATC4* up to 170kb for *NFATC2* (Fig. 1). The NFAT genes are conserved in their central regions but are less similar in the 5′ and 3′ parts. The identity on the nucleotide level and the identity and strong similarity of the amino acids on the protein level among the most conserved part of the genes, encoded by exons V–VII, is ∼ 80%. Although these exons are strongly similar, other exons are not so conserved. The identity among the full-length NFAT coding regions is ∼ 50% on the nucleotide level and the amino acid identity or strong similarity in sum of the human NFAT proteins is ∼ 56%.

**Supplementary Fig. 1:**
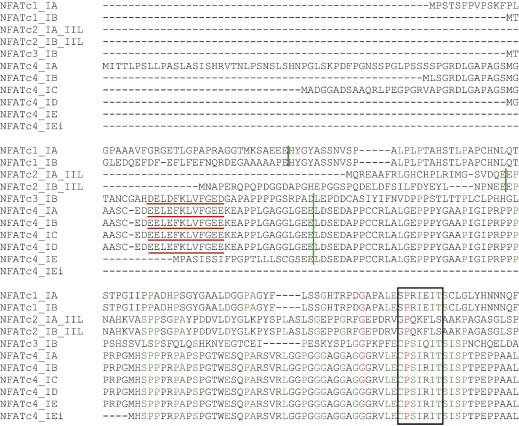
Aligment of the human N-terminal regions of the NFAT protein isoforms translated from alternative splice variants compiled with MULTALIN (*http://npsa-pbil.ibcp.fr/cgi-bin/npsa_automat.pl?page=npsa_multalin.html*). Red characters indicate identical amino acids and green characters indicate amino acids conserved 50% - 90 % among all the sequences aligned. Blue characters indicate similar amino acids between all the sequences aligned. The conserved N-terminal calcineurin binding site in the regulatory domain is highlighted with a black box. Stretch of 12 aa conservation is indicated with a red underline. Vertical green lines denote the start of exon II encoded sequences.

**Supplementary Fig. 2:**
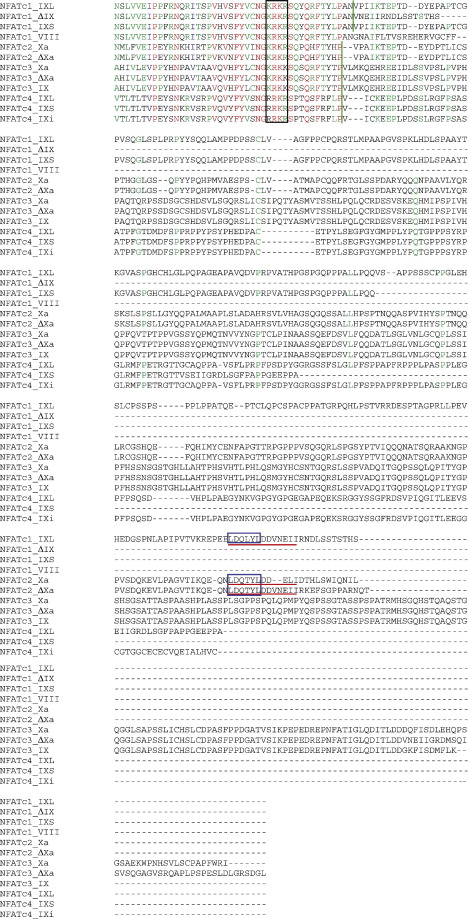
Aligment of the human C-terminal regions of the NFAT protein isoforms translated from alternative splice variants compiled with MULTALIN (*http://npsa-pbil.ibcp.fr/cgi-bin/npsa_automat.pl?page=npsa_multalin.html*). Red characters indicate identical amino acids and green characters indicate amino acids conserved 50% - 90 % among all the sequences aligned. Blue characters indicate similar amino acids between all the sequences aligned. The conserved C-terminal nuclear localisation signal (NLS) is highlighted with a black box. Conserved stretch of 13 amino acids containing a nuclear export signal (NES, blue box) is indicated with a red underline. Vertical green lines denote the end of exon VIII encoded sequences.

There are several alternative transcripts for the NFAT genes, which are generated by usage of alternative 5′ and 3′ exons and alternative splicing. According to our data, human and mouse *NFATC1* and *NFATC2,* and mouse *Nfatc3* and *Nfatc4,* have two alternative 5′ exons. In human we detected six alternative 5′ exons for *NFATC3* and seven alternative 5′ exons for *NFATC4.* Our results also showed that in both human and mouse, *NFATC1* and *NFATC3* have two alternative 3′ exons and *NFATC4* has one 3′ exon. For *NFATC2* we detected one 3′ exon in human but three alternative 3′ exons in mouse. Due to these differences, the amino acid sequences within the C- and N-termini of different NFAT protein isoforms are distinct (Supplementary Figs. 1 and 2). In this study the alternative transcripts and protein isoforms have been given names according to the alternative exons used in the respective mRNAs (Fig. 1).

### Alternative splicing and expression of NFATC1 in human and mouse

For both human and mouse *NFATC1,* there are two alternative 5′ exons, exons IA and IB, and two alternative 3′ exons, exons VIII and X (Fig. 1; Table 1). Also, in both human and mouse exon IX has two alternative splice variants, designated here IXL and IXS, which are generated by the usage of alternative splice donor sites. In addition, we detected a novel splice variant for human *NFATC1* lacking exon IX, indicated here as ΔIX. In human, exon IA encodes 42 amino acids (aa). Exon IB, located downstream from exon IA, encodes 29 aa. Transcripts that have the polyadenylation signal in exon VIII (VIII3′UTR) encode 63 aa from exon VIII, whereas transcripts containing exon X as the 3′ exon encode a C-terminal region that includes 44 aa identical to the exon VIII3′UTR isoforms in the region encoded by exon VIII, but contain an additional 245 aa or 127 aa, in the case of IXL or IXS usage, respectively. If exon IX is skipped the corresponding protein isoform lacks the 230 aa encoded by exon IXL but contains 15 C-terminal amino acids identical to the C-terminus of the exon IXL-comprising isoform. Altogether, according to our data, the possible transcript types of *NFATC1* in human are: (1) *NFATC1-IA/IB-IXL,* containing 5′ exon IA or IB, exon IXL, and 3′ exon X, having a protein coding region of 2829bp when exon IA is used or 2790bp when exon IB is used; (2) *NFATC1-IA/IB-ΔIX,* containing 5′ exon IA or IB, no exon IX, and 3′ exon X, having a protein coding region of 2139bp when exon IA is used or 2100bp when exon IB is used; (3) *NFATC1-IA/IB-IXS,* containing 5′ exon IA or IB, IXS, and 3′ exon X; exon IXS changes the open reading frame, introducing a stop codon in exon X that is 44bp upstream of the stop codon in type 1 and type 2 transcripts; the protein coding regions of *NFATC1* type 3 mRNAs are 2475bp when exon IA is used or 2436bp when exon IB is used; and (4) *NFATC1-IA/IB-VIII,* containing 5′ exon IA or IB and 3′ exon VIII; the coding region of exon VIII in type 4 transcripts is 56bp longer than in type 1, 2, and 3 transcripts; the protein coding regions are 2148bp when exon IA is used or 2109bp when exon IB is used (Fig. 1 and Supplementary Table 1). A human EST sequence corresponding to the *NFATC1* transcript lacking exon II is present in the databases; however, we did not detect it with RT-PCR and there are no references in the literature to confirm the generation of this transcript. According to our data *Nfatc1* transcripts for mouse are the same as in human, except for differences in the lengths of exons II, III, IXL, and IXS and in the length of the protein coding region of exon IB (data not shown).

**Fig. 2:**
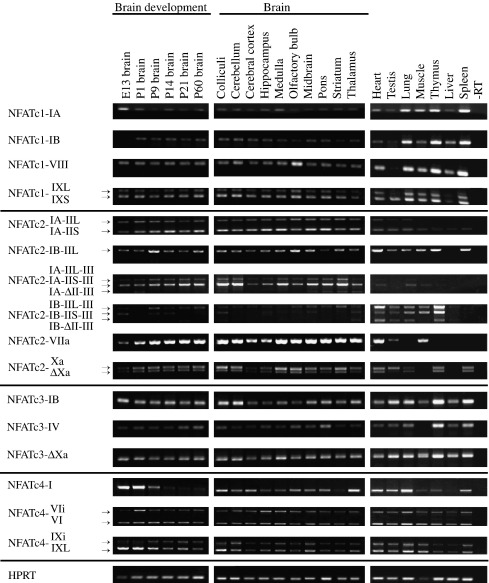
Semiquantitative analysis of *Nfatc1, Nfatc2, Nfatc3, Nfatc4,* and control *Hprt* mRNA expression by RT-PCR in different mouse brain regions, in mouse brain at the indicated developmental time points, and in various mouse tissues.

In mouse, *Nfatc1* transcripts containing exon IA or exon IB were both predominantly expressed in the lung, thymus, and spleen (Fig. 2). Both alternative 3′ exon transcripts were detected in all tissues analyzed, with the levels being highest in the lung, thymus, and spleen. Exon IXS was more abundantly used than exon IXL in 3′ exon X-containing transcripts. In the adult mouse brain and during postnatal development of the brain the expression levels of transcripts containing exon IA or exon IB were similarly low (Fig. 2). In embryonic mouse brain, only *Nfatc1* transcripts comprising exon IA were expressed. In all the brain regions tested the expression levels of both of the 3′ exon transcripts were lower compared to the levels of respective mRNAs in the thymus, spleen, or lung (Fig. 2).

**Fig. 3:**
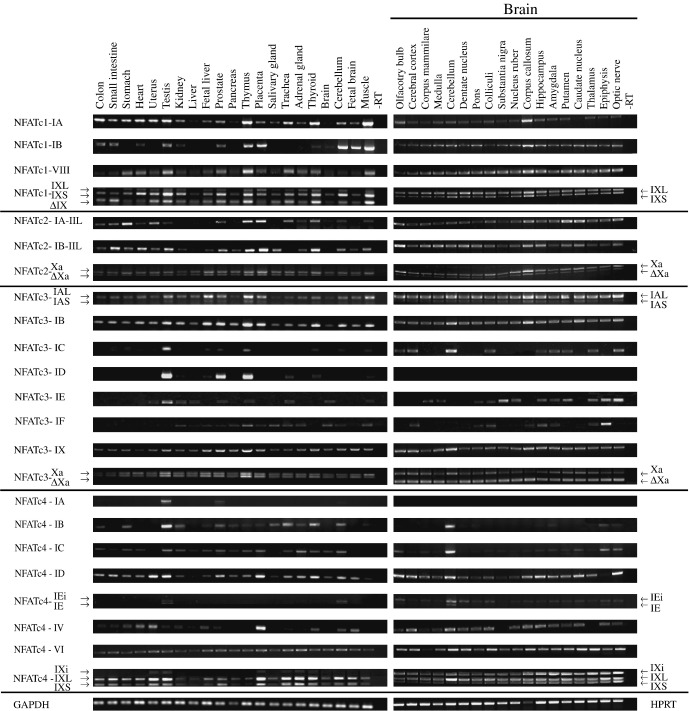
Semiquantitative analysis of *NFATC1, NFATC2, NFATC3, NFATC4,* and controls *GAPDH* and *HPRT* mRNA expression by RT-PCR in various human tissues and brain regions.

In human, *NFATC1* transcripts comprising exon IA were expressed more widely than transcripts comprising exon IB (Fig. 3). Both 5′ exon transcripts were highly expressed in the thymus and muscle. In addition, exon IA transcripts were expressed at high levels in the colon, small intestine, stomach, heart, uterus, testis, and thyroid and were detected also in other tissues. Exon IB mRNAs were highly expressed also in the fetal brain, cerebellum, and placenta and were undetectable in the stomach, uterus, liver, fetal liver, pancreas, salivary gland, trachea, and adrenal gland. Both 3′ exon transcripts were expressed in all the tissues analyzed, with highest levels observed in the testis, thymus, and muscle. Transcripts containing the 3′ exon X and exon IXS were expressed more predominantly than transcripts containing exon IXL or those in which exon IX was skipped. mRNAs with exon IXL were expressed at significantly lower levels, particularly in the small intestine, heart, liver, pancreas, salivary gland, brain, and fetal brain. Transcripts lacking exon IX or containing exon IXS were both highly and at comparable levels expressed in the thymus, thyroid, and muscle. The analysis of the expression of *NFATC1* transcripts in various human brain regions showed that transcripts comprising exon IA or exon IB were present in all regions tested, with highest levels in the corpus callosum (Fig. 3). Exon IA transcripts were expressed at relatively higher levels also in the olfactory bulb. Both of the alternative 3′ exons were also expressed in all of the regions analyzed, with the highest levels in the corpus callosum. The relative ratio of transcripts containing IXL or IXS was similar in most of the regions analyzed, except in the olfactory bulb, cerebral cortex, corpus mammilare, medulla, pons, and substantia nigra, where the expression levels of transcripts containing IXL were slightly higher. Transcripts lacking exon IX were not detected in the brain (Fig. 3).

### Alternative splicing and expression of NFATC2 in human and mouse

Both human and mouse *NFATC2* contain two alternative 5′ exons, IA and IB, whereas only exon IB has been previously described (Fig. 1 and [41]). There is one 3′ exon in human, exon X, and three 3′ exons in mouse, exons III, VIIa, and X (Table 1). Due to the usage of alternative splice acceptor sites exon II has two splice variants—IIL and IIS. Exon IIL is 1030 or 1036bp in length in human or mouse, respectively, and exon IIS is 516bp in both organisms. The shorter splice variant of exon II has not been described before. In mouse, exons IIS and IIL are both used and transcripts without exon II are also expressed, whereas in human the transcripts containing IIL are predominant and the usage of exon IIS or skipping of exon II is barely detectable. In both human and mouse exon IA encodes 23 aa and exon IB encodes 43 aa. Regardless of the 5′ exon, the translation start codon is positioned in exon II when exon IIS is used in transcripts. This leads to N-terminally truncated isoforms that are 199 and 201 aa (IA) or 219 and 221 aa (IB) shorter than the isoforms encoded by transcripts containing exon IIL in human and mouse, respectively. In both human and mouse *NFATC2* there is an additional exon compared to *NFATC1,* located upstream of exon X, named Xa here, that is either spliced in the mRNA or skipped. Usage of exon Xa leads to a translation stop codon in exon Xa. If exon Xa is not used then the translation stop codon is in exon X. This generates unique C-terminal sequences of 17 or 13 aa, for the respective NFATC2 protein isoforms. Taken together, our results showed that the possible transcript types of *NFATC2* in human are: (1) *NFATC2-IA/IB-IIL-Xa,* containing 5′ exon IA or IB, exon IIL, exon Xa, and 3′ exon X, having a protein coding region of 2703bp when exon IA is used or 2763bp when exon IB is used; (2) *NFATC2-IA/IB-IIS-Xa,* containing 5′ exon IA or IB, exon IIS, exon Xa, and 3′ exon X, having a protein coding region of 2106bp with either exon IA or exon IB; (3) *NFATC2-IA/IB-IIL-ΔXa,* containing 5′ exon IA or IB, exon IIL, and 3′ exon X and lacking exon Xa, having a protein coding region of 2715bp when exon IA is used or 2775bp when exon IB is used; (4) *NFATC2-IA/IB-IIS-ΔXa,* containing 5′ exon IA or IB, exon IIS, and 3′ exon X and lacking exon Xa, having a protein coding region of 2118bp with either exon IA or exon IB (Supplementary Table 1).

There are several differences in the mouse *Nfatc2* transcripts compared to the human ones in addition to the length of exon II. First, there are three alternative 3′ exons in mouse: exon III, exon VIIa, and exon X. The proteins encoded by transcripts using exon III or VIIa as 3′ exon lack the whole or a part of the Rel homology domain, respectively. Second, in mouse we detected *Nfatc2* transcripts lacking exon II (ΔII). Of note, according to the mouse EST and mRNA data in the NCBI databases there are transcripts in which exon V is used as the 3′ exon and transcripts with an alternative 5′ exon between exons III and IV. However, we did not detect these transcripts with PCR and there are no references to these transcripts in the literature.

Mouse *Nfatc2* transcripts containing exon IA, including transcripts with the mouse-specific 3′ exon III and transcripts with the 3′ exon VIIa, were all highly expressed in the brain, where the expression levels increased during postnatal development. In the adult mouse brain high levels were observed in the colliculi, cerebellum, medulla, olfactory bulb, and striatum (Fig. 2). Exon VIIa transcripts were highly expressed also in the heart and muscle. The levels of transcripts with exon X as the 3′ exon, either containing or lacking exon Xa, were relatively higher in the heart, testis, thymus, spleen, and brain, where they were more abundant in the colliculi, cerebellum, medulla, olfactory bulb, and striatum. Transcripts including exon IB predominantly contained exon IIL and were more broadly expressed, with relatively higher expression levels in the heart, thymus, and spleen. Transcripts containing exons IB and III3′UTR showed the highest expression levels in the heart and thymus and were very weakly expressed in the brain (Fig. 2).

In human, *NFATC2* transcripts including exon IA spliced to exon IIL were highly expressed in the stomach, uterus, thymus, placenta, trachea, and thyroid (Fig. 3). Transcripts containing exons IB and IIL were expressed highly in the small intestine, heart, testis, prostate, thymus, placenta, and thyroid. Transcripts containing IIS were hardly detectable (data not shown). Transcripts of *NFATC2* containing the 3′ exon X and either comprising or lacking exon Xa (ΔXa) were both present in all of the tissues analyzed, with exon Xa-containing transcripts being expressed at slightly higher levels. In the brain *NFATC2* was widely expressed. The expression levels were highest in the caudate putamen for exon IA and the olfactory bulb and corpus callosum for exon IB transcripts. Similar levels of exon Xa and ΔXa transcripts were expressed in almost all regions of the human brain except in the olfactory bulb, colliculi, nucleus ruber, corpus callosum, and caudate nucleus, where exon Xa transcripts were predominant (Fig. 3).

### Alternative splicing and expression of NFATC3 in human and mouse

The human *NFATC3* contains six 5′ exons located within ∼ 4500bp in the genome (Fig. 1), whereas in mouse there are only two alternative 5′ exons in *Nfatc3.* The human exon IB has been described before [23]. However, exons IA, IC, ID, IE, and IF are first described in this study (Table 1). Exon IB in human is homologous to exon IB in mouse and encodes 34 N-terminal amino acids of the respective proteins. Mouse 5′ exon IA is not homologous to human exon IA and encodes a unique N-terminus of 26 aa. In human, exon IB is the only 5′ exon that is a protein-coding exon. Exons IA, IC, ID, IE, and IF all lack an in-frame translation start codon and translation of *NFATC3* transcripts containing these exons could start from the ATG located in exon IV. Thus, proteins encoded by these transcripts would not contain the 485 N-terminal amino acids present in NFATC3 isoforms translated from transcripts including exon IB. Exon IA in human contains two alternative splice donor sites and the respective splice variants are named here IAL and IAS. There are two alternative 3′ exons in *NFATC3* genes: exons IX and X in human and exons IV and X in mouse. Transcripts in which exon IX is used as the 3′ exon encode proteins with 9 unique C-terminal amino acids that are not present in the proteins encoded by exon X-containing transcripts. Like in human *NFATC2* there is an alternative exon located between exon IX and exon X, designated here Xa, in both human and in mouse *NFATC3* genes. If exon Xa is used in the transcripts a stop codon is introduced. Therefore, different C-terminal sequences of 32 or 39 aa are encoded by exon X3′UTR transcripts depending on the usage or skipping, respectively, of exon Xa. Altogether, according to our data, the possible transcript types of *NFATC3* in human are: (1) *NFATC3-IB-Xa,* containing 5′ exon IB, exon Xa, and 3′ exon X, having a protein coding region of 3204bp; (2) *NFATC3-IB-ΔXa,* containing 5′ exon IB and 3′ exon X and lacking exon Xa, having a protein coding region of 3225bp; (3) *NFATC3-IB-IX,* containing 5′ exon IB and exon IX as the 3′ exon, having a protein coding region of 3135bp; (4) *NFATC3-IAL/IAS/IC/ID/IE/IF-Xa,* containing 5′ exon IAL or IAS or IC or ID or IE or IF, exon Xa, and 3′ exon X, having a protein coding region of 1767bp; (5) *NFATC3-IAL/IAS/IC/ID/IE/IF-ΔXa,* containing 5′ exon IAL or IAS or IC or ID or IE or IF and 3′ exon X and lacking exon Xa, having a protein coding region of 1788bp; and (6) *NFATC3-IAL/IAS/IC/ID/IE/IF-IX,* containing 5′ exon IAL or IAS or IC or ID or IE or IF and exon IX as the 3′ exon, having a protein coding region of 1698bp (Supplementary Table 1). Masuda et al. have described a human *NFATC3* transcript containing an additional exon upstream of exon X and downstream of exon Xa [26]. However, we were not able to detect this transcript in any of the tissues analyzed in this study.

In mouse, the expression of *Nfatc3* transcripts containing exon IB was detected in all tissues analyzed, with relatively higher levels in the testis, lung, thymus, and spleen (Fig. 2). Exon IA transcripts were barely detectable only in the testis and thymus (data not shown). *Nfatc3* transcripts containing the mouse-specific 3′ exon IV were expressed at moderate levels in the thymus and spleen. However, with the exception of muscle, low levels of this transcript were seen in all the tissues analyzed. Transcripts containing the 3′ exon X were also observed in all the tissues analyzed, with higher levels in the testis, lung, thymus, and spleen. In the mouse brain exon IB transcripts were expressed in all the regions analyzed (Fig. 2). Higher levels were seen in the colliculi and cerebellum. During mouse brain development the levels of *Nfatc3* transcripts containing exon IB remained unchanged from embryonic day 13 (E13) up to adult, the developmental period studied here. Transcripts with 3′ exon X were expressed in all brain regions analyzed with, ΔXa transcripts being the predominant mRNAs (Fig. 2).

In human, *NFATC3* transcripts comprising exon IA or IB were more widely expressed than exon IC, ID, IE, or IF transcripts (Fig. 3). Both IA and IB transcripts were expressed at relatively higher levels in the testis, fetal liver, thymus, and muscle. In addition, exon IB was highly expressed in the prostate, placenta, thyroid, and cerebellum. IAL-containing transcripts were expressed at higher levels than IAS-containing transcripts. Transcripts containing exon IC were highly expressed in the testis. Transcripts containing exon ID had high expression in the testis, prostate, and thymus and lower expression in the kidney, liver, fetal liver, trachea, thyroid, and muscle. Exon IE was most strongly expressed in the testis and exon IF in the thymus. All the *NFATC3* 3′ exon transcript variants were expressed in all the tissues analyzed, with higher levels detected in the testis, fetal liver, prostate, thymus, and placenta. In the heart and muscle, transcripts containing exon Xa were expressed at slightly higher levels than transcripts lacking exon Xa. In the human brain, transcripts comprising exons IA and IB were expressed in all the regions analyzed, whereas exon IC transcripts were expressed only in some regions, with relatively higher levels in the cerebellum (Fig. 3). Exon ID transcripts were not detected in the human brain. Exon IE was relatively more expressed in the substantia nigra, optic nerve, and epiphysis and exon IF in the epiphysis. Expression levels of both of the alternative 3′ exons were similar in all the regions analyzed. Compared to the other brain regions exon IX transcripts were present at slightly higher levels in the olfactory bulb, cerebral cortex, cerebellum, and corpus callosum. Transcripts containing exon Xa were mostly expressed at higher levels than transcripts lacking exon Xa (Fig. 3).

### Alternative splicing and expression of NFATC4 in human and mouse

Before our study, only one exon, named here exon ID, had been described as a 5′ exon in human *NFATC4* [23]. Our data show that human *NFATC4* has five 5′ exons within ∼ 2.5kb of the most upstream part of the gene, named here IA, IB, IC, ID, and IE. In addition, 5′-extended exons IV and VI are also used as 5′ exons (Fig. 1 and Table 1). For mouse *Nfatc4* we identified two 5′ exons: the previously described 5′ exon, named exon I here, which is homologous to the human exon ID [26], and the 5′-extended exon VI, which has not been described before. With the usage of the 5′-extended exon VI in mouse, exclusion and retention of the intron between exons VI and VII were detected. Human exon ID and mouse exon I encode 33 aa. The human exon ID is in addition used as an internal exon: 129bp of its 3′ part are always inserted as the second exon in the transcripts starting upstream of exon ID. This is due to a cryptic splice acceptor site inside exon ID. Human *NFATC4* transcripts that use the 5′ exon IA, IB, or IC encode proteins with an additional 63, 13, or 32 aa, respectively, in their N-termini compared to the protein encoded by the transcripts containing exon ID as the 5′ exon. If exon IEi (retention of intron between exons IE and II), exon IV, or exon VI is used as the 5′ exon, the corresponding human *NFATC4* transcripts encode protein isoforms that are 70, 465, or 711 aa, respectively, shorter in their N-terminus compared to the protein encoded by the transcripts containing exon ID as the 5′ exon. Exon X is used as the 3′ exon in all mouse and human *NFATC4* mRNAs (Fig. 1). In human, there are two splice variants of exon IX, named here IXL and IXS, due to the usage of alternative splice donor sites. In addition, retention of the intron between exons IX and X leads to transcript variants indicated by IXi here. If exon IXS is used, the respective protein isoforms lack 108 aa in the C-terminal region compared to protein isoforms encoded by exon IXL-containing transcripts. IXi usage leads to protein isoforms with 20 unique amino acids in the C-terminus. In mouse, only exons IXL and IXi are used. Taken together, our results showed that the possible transcript types of *NFATC4* in human are: (1) *NFATC4-IA-IXL,* containing 5′ exon IA, exon IXL, and 3′ exon X, having a protein coding region of 2895bp; (2) *NFATC4-IA-IXS,* containing 5′ exon IA, exon IXS, and 3′ exon X, having a protein coding region of 2571bp; (3) *NFATC4-IA-IXi,* containing 5′ exon IA, exon IXi, and 3′ exon X, having a protein coding region of 2892bp; (4) *NFATC4-IB-IXL,* containing 5′ exon IB, exon IXL, and 3′ exon X, having a protein coding region of 2745bp; (5) *NFATC4-IB-IXS,* containing 5′ exon IB, exon IXS, and 3′ exon X, having a protein coding region of 2421bp; (6) *NFATC4-IB-IXi,* containing 5′ exon IB, exon IXi, and 3′ exon X, having a protein coding region of 2742bp; (7) *NFATC4-IC-IXL,* containing 5′ exon IC, exon IXL, and 3′ exon X, having a protein coding region of 2802bp; (8) *NFATC4-IC-IXS,* containing 5′ exon IC, exon IXS, and 3′ exon X, having a protein coding region of 2478bp; (9) *NFATC4-IC-IXi,* containing 5′ exon IC, exon IXi, and 3′ exon X, having a protein coding region of 2799bp; (10) *NFATC4-ID-IXL,* containing 5′ exon ID, exon IXL, and 3′ exon X, having a protein coding region of 2706bp; (11) *NFATC4-ID-IXS,* containing 5′ exon ID, exon IXS, and 3′ exon X, having a protein coding region of 2382bp; (12) *NFATC4-ID-IXi,* containing 5′ exon ID, exon IXi, and 3′ exon X, having a protein coding region of 2703bp; (13) *NFATC4-IE-IXL,* containing 5′ exon IE, exon IXL, and 3′ exon X, having a protein coding region of 2670bp; (14) *NFATC4-IE-IXS,* containing 5′ exon IE, exon IXS, and 3′ exon X, having a protein coding region of 2346bp; (15) *NFATC4-IE-IXi,* containing 5′ exon IE, exon IXi, and 3′ exon X, having a protein coding region of 2667bp; (16) *NFATC4-IEi-IXL,* containing 5′ exon IEi, exon IXL, and 3′ exon X, having a protein coding region of 2496bp; (17) *NFATC4-IEi-IXS,* containing 5′ exon IEi, exon IXS, and 3′ exon X, having a protein coding region of 2172bp; (18) *NFATC4-IEi-IXi,* containing 5′ exon IEi, exon IXi, and 3′ exon X, having a protein coding region of 2493bp; (19) *NFATC4-IV-IXL,* containing exon IV as the 5′ exon, exon IXL, and 3′ exon X, having a protein coding region of 1311bp; (20) *NFATC4-IV-IXS,* containing exon IV as the 5′ exon, exon IXS, and 3′ exon X, having a protein coding region of 987bp; (21) *NFATC4-IV-IXi,* containing exon IV as the 5′ exon, exon IXi, and 3′ exon X, having a protein coding region of 1308bp; (22) *NFATC4-VI-IXL,* containing exon VI as the 5′ exon, exon IXL, and 3′ exon X, having a protein coding region of 570bp; (23) *NFATC4-VI-IXS,* containing exon VI as the 5′ exon, exon IXS, and 3′ exon X, having a protein coding region of 246bp; and (24) *NFATC4-VI-IXi,* containing exon VI as the 5′ exon, exon IX, and 3′ exon X, having a protein coding region of 567bp (Supplementary Table 1).

Expression of mouse *Nfatc4* exon I mRNA was detected in all tissues analyzed, with higher levels in the lung, heart, testis, and spleen (Fig. 2). During brain development, the highest levels were observed at E13, the first developmental stage analyzed, and the expression decreased thereafter, reaching the lowest levels at postnatal day 14 and remaining unchanged thereafter. In the adult mouse brain the highest levels of *Nfatc4* exon I transcripts were detected in the thalamus and colliculi. Transcripts containing the extended 5′ exon VI or VIi were evenly expressed in all the peripheral tissues tested (Fig. 2). The highest expression levels of *Nfatc4* exon IXL and IXi transcripts were detected in the lung, heart, and muscle. In the brain IXL transcript levels decreased during development, while IXi transcripts were higher during postnatal development. In adult mouse brain these transcripts were most strongly expressed in the colliculi, midbrain, and cerebellum (Fig. 2).

In human, *NFATC4* transcripts comprising 5′ exons IB, IC, ID, and VI were expressed more widely than 5′ exon IA, IE, and IV transcripts (Fig. 3). Exon IA transcripts were detected only in the testis and prostate. Exon IB and IC transcripts were both expressed in several tissues, including the stomach, testis, kidney, trachea, adrenal gland, thyroid, and cerebellum. Transcripts containing exon ID were expressed in almost all tissues analyzed, with the highest levels in the small intestine, uterus, testis, prostate, placenta, thyroid, and cerebellum. Low levels of exon IE- and IEi-containing transcripts were seen only in the testis. The 5′ exon IV transcript levels were highest in the placenta and relatively high also in the heart and uterus. Expression levels of transcripts containing exon VI as the 5′ exon were moderate in all tissues tested (Fig. 3). The 3′ exon X-containing transcripts of *NFATC4* were expressed according to the sum of the expression patterns of the 5′ exons. Highest levels were detected in the uterus, testis, placenta, trachea, adrenal gland, thyroid, and cerebellum. In most tissues, transcripts containing exon IXL were relatively more abundant than transcripts containing exon IXS. Transcripts containing the 3′ exon IX were expressed at low levels in the testis, kidney, and thymus. In the brain, all the transcript types of *NFATC4,* except exon IA transcripts, were expressed in the cerebellum (Fig. 3). In addition, high levels of 5′ exon ID, IV, and VI transcripts were detected in other brain regions: exon ID mRNAs in the olfactory bulb, hippocampus, caudate nucleus, and optic nerve; exon IV transcripts in the cerebral cortex, corpus callosum, and hippocampus; and exon VI transcripts in the olfactory bulb and cerebellum. Expression of exon X-containing transcripts, corresponding to the sum of all *NFATC4* mRNAs, was detected in all brain regions, with highest levels in the cerebellum, where exon IXL-containing transcripts were the predominant ones (Fig. 3).

### In situ hybridization analyses of NFATC1, NFATC2, NFATC3, and NFATC4 expression in adult mouse brain and human hippocampus

Expression of NFAT mRNAs at the cellular level has not been studied thoroughly by in situ hybridization before. Therefore we analyzed the expression of NFAT mRNAs in adult mouse brain by in situ hybridization. The hybridization probes for each NFAT mRNA were constructed to recognize all of the major splice variants and therefore were targeted to the conserved RHD coding region of NFAT mRNAs. To distinguish different cell types, Nissl counterstaining of the tissue sections, which allows one to distinguish the large and weakly stained nuclei of neurons from the small and strongly stained nuclei of glial cells, was used. All of the NFAT mRNAs were expressed in the neurons of the brain, with specific patterns for each NFAT.

**Supplementary Fig. 3:**
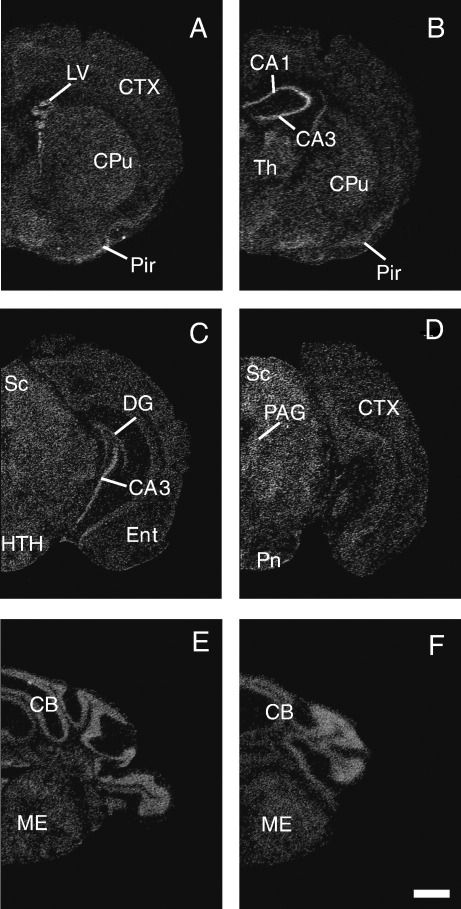
*In situ* hybridization analysis of *NFATc2* mRNA expression in adult mouse brain. Dark-field emulsion autoradiographs from coronal sections at the level of striatum (A), thalamus (B), hippocampus (C and D) and cerebellum (E and F). LV, lateral ventricle; CPu, caudate putamen; CTX, cortex; Pir, piriform cortex; Th, thalamus; CA1, field CA1 of hippocampus; CA3, field CA3 of hippocampus; Sc, superior colliculi; Ent, entorhinal cortex; HTH, hypothalamus; PAG, periaqueductal gray; Pn, pontine nuclei; DG, dentate gyrus; CB, cerebellum; ME, medulla. Scale bar is 1 mm.

**Fig. 4:**
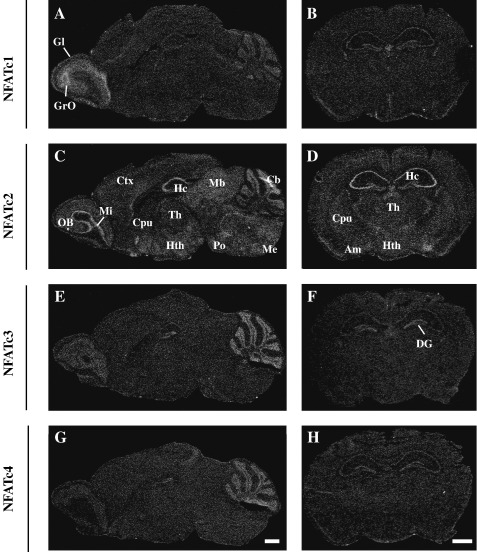
In situ hybridization analysis of *Nfatc1, Nfatc2, Nfatc3,* and *Nfatc4* mRNA expression in adult mouse brain. Dark-field emulsion autoradiographs from sagittal sections (A, C, E, and G) and coronal sections at the level of thalamus (B, D, F, and H). The sections were hybridized with a probe for *Nfatc1* (A and B), *Nfatc2* (C and D), *Nfatc3* (E and F), or *Nfatc4* (G and H). Gl, glomerular layer of olfactory bulb; GrO, granular layer of olfactory bulb; OB, olfactory bulb; Mi, mitral layer of olfactory bulb; Ctx, cortex; Cpu, caudate putamen; Hc, hippocampus; Th, thalamus; Hth, hypothalamus; Mb, midbrain; Po, pons; Cb, cerebellum; Me, medulla; Am, amygdala; DG, dentate gyrus. Scale bars, 1 mm.

**Fig. 5:**
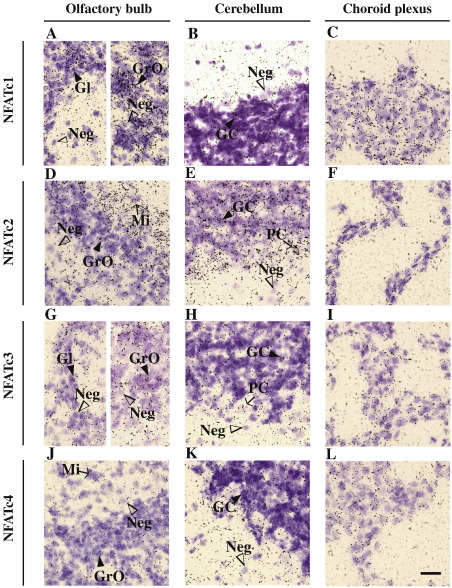
In situ hybridization analysis of *Nfatc1, Nfatc2, Nfatc3,* and *Nfatc4* mRNA expression in adult mouse brain. Bright-field higher magnification pictures of the olfactory bulb (A, D, G, and J), cerebellum (B, E, H, and K), and choroid plexus (C, F, I, and L) are shown. Filled arrowheads denote some positive neurons, some negative neurons are marked with unfilled arrowheads. The sections were hybridized with a probe for *Nfatc1* (A, B, and C), *Nfatc2* (D, E, and F), *Nfatc3* (G, H, and I), or *Nfatc4* (J, K, and L). Gl, glomerular cell layer of the olfactory bulb; GrO, granular cells of the olfactory bulb; Mi, mitral cell layer, PC, Purkinje cells; GC, granular cells of the cerebellum; Neg, negative cell. Scale bar, 50 μm.

We observed similarities and differences in the expression patterns of *Nfatc1, Nfatc2, Nfatc3,* and *Nfatc4* (Figs. 4 and 5 and Supplementary Fig. 3). In the olfactory system *Nfatc1* was highly expressed in the granular layer and glomerular cell layer and *Nfatc2* in the mitral cell layer (Figs. 4 and 5). A moderate signal was detected in the glomerular and granular cell layer for *Nfatc2* (Figs. 4A, 4C, 5A, and 5D). *Nfatc3* and *Nfatc4* were expressed at low levels in the glomerular and granular layer (Figs. 4E, 4G, 5G, and 5J). In addition, *Nfatc4* was expressed in the mitral cell layer (Figs. 4G and 5J).

In the cerebral cortex *Nfatc2* mRNA expression was detected at relatively high levels in the neurons of layers II–VI (Fig. 4C and Supplementary Figs. 3A–D). The expression levels of other NFAT family members in this brain region were below the detection limit of our in situ hybridization method. Although all NFAT mRNAs were detected in the hippocampal formation, there were differences in the distribution and level of expression between different NFAT genes. *Nfatc2* showed a strong signal in the hippocampus, particularly in the CA1–CA3 pyramidal layers, and slightly lower levels in the granular layer of the dentate gyrus (Figs. 4C and D and Supplementary Figs. 3B–D). *Nfatc3* mRNA was moderately expressed only in the granular layer of the dentate gyrus and was detected at low levels in the CA1–CA3 pyramidal layers (Figs. 4E and F). *Nfatc1* and *Nfatc4* mRNAs were expressed at evenly low levels in both the CA1–CA3 pyramidal cells and the dentate gyrus granular cells (Figs. 4A and B).

In the basal ganglia, evenly distributed moderate signal was detected for *Nfatc2* in the caudate putamen, ventral pallidum, accumbens, and septum (Fig. 4C and Supplementary Fig. 3A), whereas *Nfatc1* mRNA was expressed at moderate levels only in the region of bed nucleus of stria terminalis (data not shown). *Nfatc3* and *Nfatc4* mRNAs were not expressed significantly in these brain structures (Figs. 4E and G). In the thalamus, hypothalamus, and midbrain *Nfatc2* mRNA was widely expressed at high or moderate levels in most of the nuclei (Figs. 4C and D and Supplementary Figs. 3B and C). In contrast, signal for *Nfatc1, Nfatc3,* and *Nfatc4* was barely detectable in these structures (Figs. 4A, B, and E–H).

In the cerebellum all NFAT transcripts were expressed (Figs. 4 and 5). *Nfatc1* and *Nfatc4* were moderately expressed only in the granular neurons (Figs. 4A, 4G, 5B, and 5K), whereas *Nfatc2* and *Nfatc3* were expressed in the granular cell layer and also in the Purkinje cell layer (Figs. 4C, 4E, 5E, and 5H). In the granular layer cells the signal for *Nfatc2* and *Nfatc3* was relatively stronger than that for *Nfatc1* and *Nfatc4*. In the pons and medulla only *Nfatc2* showed moderate expression all over the region (Fig. 4C and Supplementary Figs. 3E and F).

In the choroid plexus and ependymal cells *Nfatc1* and *Nfatc2* were expressed at high levels and *Nfatc3* and *Nfatc4* at moderate levels (Figs. 5C, F, I, and L).

**Fig. 6:**
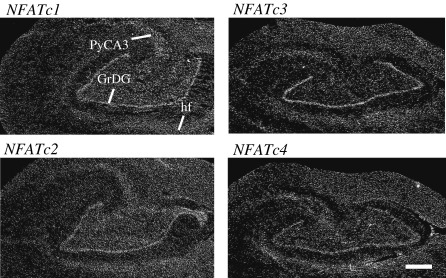
In situ hybridization analysis of *NFATC1, NFATC2, NFATC3,* and *NFATC4* mRNA expression in adult human hippocampus. Dark-field emulsion autoradiographs from coronal sections at the level of hippocampus. GrDG, granular cell layer of the dentate gyrus; PyCA3, CA3 pyramidal cells; hf, hippocampal fissure. Scale bar, 1 mm.

In the human hippocampus all four NFATs were expressed. For all NFATs stronger signal was detected in the granular layer of the dentate gyrus, in the pyramidal neurons of CA3 region, and in the hippocampal fissure (Fig. 6).

## Discussion

The aim of this study was to characterize the structures, alternative splicing, and expression of the NFAT genes in human and in mouse. Our results on the structures of the NFAT genes are in agreement with previous data from other groups [28,29,33,42,43] and also add important new data about the complex splicing and expression of this gene family. NFAT genes encode proteins that are very similar in their central region, which encodes the Rel homology domain, but are clearly variable in their N- and C-terminal parts due to the less conserved 5′ and 3′ regions of the paralogs of the genes, which differ significantly in their protein-coding potencies. Here we show that the NFAT genes are even more diverse in the 5′ and 3′ regions than previously described. All NFAT genes in human and mouse have multiple alternatively used 5′ exons: according to our data human and mouse *NFATC1* and *NFATC2* and mouse *Nfatc3* and *Nfatc4* have two alternative 5′ exons; human *NFATC3* has six and *NFATC4* has seven alternative 5′ exons. We show that usage of 3′-terminal exons is also complex: human and mouse *NFATC1* and *NFATC3* genes contain two and the mouse *Nfatc2* contains three alternative 3′ exons. Human *NFATC2* and both human and mouse *NFATC4* have one 3′ exon. In addition, alternative splicing is used for all NFAT genes and in combination with the usage of alternative 5′ and 3′ exons this could theoretically lead to 8 different protein isoforms of NFATC1, 6 different isoforms of both NFATC2 and NFATC3, and 24 different isoforms of NFATC4 in human.

The alternative 5′ exons of *NFATC1* have been described before [24,29,44,45] and the results of our bioinformatic and expression studies confirm that the *NFATC1* gene has two alternative 5′ exons in both mouse and human. Also, our expression analyses are consistent with studies showing *NFATC1* expression predominantly in the immune system [23–26,46]. For *NFATC2* only one 5′ exon had been previously described [41]. Here we have identified and characterized a novel alternative 5′ exon for both human and mouse *NFATC2*. We show that in human the previously known *NFATC2* 5′ exon, exon IB, and the novel alternative 5′ exon, named here exon IA, are expressed at similar levels in most of the tissues, except in the heart, where exon IB-containing mRNAs are the predominant transcripts. In mouse though, exon IA is used predominantly in the brain and exon IB both in the brain and in nonneural tissues. In addition to the novel *NFATC2* 5′ exon we describe a novel splice variant of exon II that is conserved in human and mouse and results in a short form of exon II, named IIS here. Usage of the short variant of exon II is the predominant splicing event in mouse for transcripts starting with exon IA. However, in human, exon IIS transcripts of *NFATC2* are barely detectable. *NFATC2* exon IIS-containing transcripts encode proteins without the N-terminal CaN binding site and serine-rich region. The functions of such isoforms are yet to be elucidated.

We have shown here that human *NFATC3* and *NFATC4* contain six and five 5′ exons, respectively. The human *NFATC3* exon IB and *NFATC4* exon ID are the only 5′ exons homologous to a 5′ exon in mouse *Nfatc3* and *Nfatc4,* respectively. These homologous exons are the only 5′ exons that have been previously described for *NFATC3* and *NFATC4* [23]. In human *NFATC3* and *NFATC4* the 5′ exons are located very close to each other in the 5′ ends of the genes. The close placement of the exons leaves open the possibility that they compile one exon with multiple transcription start sites. However, bioinformatic and PCR analyses showed that the 5′ sequences of both human *NFATC3* and *NFATC4* transcripts are not overlapping and use distinct splice donor sites, indicating that they are derived from different exons. In addition, there is an alternative transcription start site in human *NFATC4* located upstream of exon IV. Usage of this transcription start site generates protein isoforms that are similar to the *NFATC3* isoforms encoded by transcripts with the novel 5′ exons IA, IC, and ID of *NFATC3,* which yield NFAT isoforms without the whole regulatory domain. The function of these proteins is unclear. However, since we found generation of such isoforms for both *NFATC3* and *NFATC4* in human, these isoforms could be important in functions yet undefined. Another interesting observation about the putative N-termini of the human *NFATC3* and *NFATC4* is that although exon IB of human *NFATC3* and exons IA, IB, IC, and ID of human *NFATC4* encode N-termini that are different comparing the whole sequence, they clearly contain a conserved stretch of 12 amino acids—[E/D]EL[E/D]FKLVFGE[E/D] (Supplementary Fig. 1). The N-termini of the NFAT proteins have previously been shown to contain TADs [17]. We suggest that the TADs of the NFAT proteins contain important motifs that probably function similarly but have also evolved to be distinct, potentially to convey different gene regulation activities.

The 3′ regions of NFAT genes are also diverse due to alternative splicing and usage of alternative 3′ exons. For example, in human, exon IX of *NFATC1* can be alternatively spliced in three ways, leading to transcripts lacking exon IX (ΔIX), with a long variant of exon IX (IXL) or with a short variant of exon IX (IXS). ΔXI transcripts have not been described before. Similar to the N-termini of NFAT proteins, there is a conserved motif (LDQ[T/L]YLDD(VN)E[I/L]I[R/D]) in the C-terminal sequences that locates to the C-terminal TAD of the NFATC1 and NFATC2 proteins [17] (Supplementary Fig. 2). This sequence is present only in the NFATC1 isoform NFATC1-IXL, whereas it is present in all human NFATC2 isoforms (Supplementary Fig. 2). In addition, by bioinformatics we found that this conserved motif contains a putative NES (Supplementary Fig. 2). Hence the isoforms lacking the sequence may act differently compared to the isoforms containing the TAD/NES. Our expression analysis of *NFATC1* showed that splice variants with exon IXS, encoding protein isoforms lacking the conserved motif, are the most abundantly expressed transcripts.

Alternative 3′ exons of NFAT genes are used in a species-specific manner. For example, for the mouse *Nfatc2,* exon VIIa has been shown to be an alternative 3′ exon and transcripts that include this exon are expressed specifically in neurons and encode a constitutively active isoform [33]. Our expression analysis confirmed that this *Nfatc2* transcript, named here *Nfatc2IB-IIS-VIIa,* is expressed in the mouse brain. However, in human, the homolog of this *Nfatc2* transcript is not expressed. Our analysis showed that similar species-specific differences are present also for human and mouse *NFATC3.* In human, and not in mouse, exon IX is used as an alternative 3′ exon. Transcripts including this exon encode the human-specific NFATC3 protein isoform containing a C-terminal TAD with a different transactivation potential compared to other NFATC3 C-terminal TADs [28]. In this study we have shown that this transcript is expressed in all human tissues analyzed. Furthermore, we show that *Nfatc3* transcripts using exon IV as the 3′ exon encoding a protein isoform lacking most of the Rel homology domain are expressed in mouse, in most of the tissues analyzed, and not in human. These data combined show that different NFAT C-termini could potentially have functionally unique characteristics, emphasizing that although NFAT proteins may have redundancy in their functions, as can be concluded from knockout studies [47,48], the specific functions of different NFAT isoforms could have an impact on Ca^2+^-dependent gene expression in different tissues and species depending on the isoform expressed. Species-specific splicing and usage of alternative 5′ exons have also been shown to regulate other genes, for example, splicing of the *p53* tumor suppressor gene [49] and usage of 5′ exons of the transcription factor *NR5A1* [50].

In previous studies only *NFATC4* expression has been characterized in more detail in some regions of adult nervous system and cultured primary neurons [21,35,36,38,39,51,52]. However, there is evidence that NFATC2 and NFATC3 might also have important functions in the nervous system. Mice with the combination of *Nfatc2, Nfatc3,* and *Nfatc4* mutations have a complete defect of midline crossing of the commissural neurons in response to netrins and neurons from these mice are unable to respond to neurotrophins [47], whereas NFAT single-knockout mice have lesser neuronal defects [2,19]. In addition, there is evidence that *NFATC2* and *NFATC3* are expressed in some regions of the brain—in the hypothalamus and olfactory bulb [24,31,32]. To date there are no indications of *NFATC1* expression in the brain. To find out which of the NFAT genes are expressed in the adult brain and in which cells they are expressed we analyzed expression of NFAT mRNAs in adult mouse brain using in situ hybridization. Our results showed that *Nfatc2* is broadly expressed throughout the adult mouse brain and is the predominantly expressed NFAT in the mouse brain. The highest *Nfatc2* mRNA levels were detected in the pyramidal cell layer of the CA1–CA3 regions of the hippocampus, in the Purkinje and granule cell layers of the cerebellum, olfactory bulb, hypothalamus, and thalamus. Surprisingly, we found that *Nfatc1* is also expressed in mouse brain, having the highest expression levels in the granular and glomerular cell layers of the olfactory bulb. Moderate expression was seen also in the cerebellar granule cells. This suggests that NFATC1 may have important functions in the brain in addition to the functions in the tissues described earlier by others [24,26,52]. Before this study *NFATC3* expression in the brain had been demonstrated only in the hypothalamus and striatum and in certain cell lines of neuronal origin [31,35]. Here we describe *NFATC3* expression in the cerebellar granule cells and in Purkinje cells and, to a lesser extent, in the granule cells of the dentate gyrus and olfactory bulb. Therefore, our findings are consistent with the knockout studies showing that both *NFATC2* and *NFATC3* contribute to the development and function of the nervous system [47].

Several studies have shown that *NFATC4* is expressed in the brain, giving the notion that *NFATC4* might be the most abundant NFAT expressed in the nervous system. Expression has been shown in the adult rat hippocampus using RT-PCR and in situ hybridization and in the adult mouse hippocampus using RT-PCR [21], in cultured rat striatal cells using Western blot analysis [35] and in the adult mouse striatum using immunohistochemistry [51], in adult mouse dorsal root ganglion and spinal cord using in situ hybridization [36], in cultured rat cerebellar granule cells using immunocytochemistry [38], in adult mouse hippocampus using immunohistochemistry [39], and in cultured rat hippocampal neurons using immunocytochemistry [53]. However, we detected only low levels of *Nfatc4* expression in the brain. To ensure credibility we repeated the assay twice using two different hybridization probes. We show here that *Nfatc4* is indeed expressed in the brain but at lower levels compared to the other NFATs. Moderate expression was detected only in the cerebellar, olfactory bulb, and dentate gyrus granule cells and in the mitral cells of the olfactory bulb. On the other hand, according to our PCR analysis of NFAT expression during mouse brain development, *Nfatc4* was more abundantly expressed in the earlier stages of development and the levels were decreased during later stages. Therefore, Nfatc4 function might be especially important in the developing brain. Surprisingly, our PCR results showed that *NFATC4* is relatively highly expressed in the human brain, indicating again that NFAT signaling could have species-specific features. Moreover, in situ hybridization analysis showed that in the adult human brain all NFAT genes are expressed in the CA1–CA3 pyramidal neurons and dentate gyrus granule cells.

Notably, in the human brain all the NFATs, among other regions, were expressed in the corpus callosum and optic nerve—regions of the brain containing mainly neuronal projections and glial cells. By in situ hybridization, we found that the majority of the signal in the mouse brain accumulated in certain neuronal populations, although for all NFATs, very low signal was detected all over the brain, including glial cells. Thus, it is possible that in addition to neurons, NFAT genes are also expressed in glial cells. In the human hippocampus all NFATs were expressed in the CA3 pyramidal neurons and dentate gyrus granular cell layer and also in the hippocampal fissure, which further supports the possibility that NFAT genes are expressed in both neurons and glial cells.

In conclusion, the results of this study provide comprehensive data about the structure, splicing pattern, and expression of NFAT genes in human and mouse, providing useful information for future studies aiming to elucidate the functions of different NFAT isoforms.

## Materials and methods

### Structure, expression analysis, and cloning of the NFAT genes

Human NFAT gene structures and human and mouse NFAT mRNAs were identified by analyzing genomic, mRNA, and EST databases using bioinformatics tools (http://www.ncbi.nlm.nih.gov and http://genome.ucsc.edu). All homology searches were performed using various BLAST tools (http://www.ncbi.nlm.nih.gov). NESs were predicted using the software at http://www.cbs.dtu.dk/services/NetNES/ site and NLSs were predicted using software at http://wolfpsort.org/ site. Rel homology and serine-rich sequence motifs were predicted using the software at the http://expasy.org/prosite/ site. Based on the sequence information acquired, primers were designed to analyze the expression of human and mouse NFAT mRNAs and to construct plasmids for mouse and human NFAT riboprobe generation (Supplementary Table 2). Total RNAs from various human and mouse brain regions and mouse tissues were purified with RNAwiz (Ambion, USA) as recommended by the manufacturer and treated with DNase using a DNA-free kit (Ambion). Human RNAs from different tissues were obtained from BD Biosciences (USA). First-strand cDNAs were synthesized from 5 μg of total RNA with Superscript III reverse transcriptase (Invitrogen, USA) as recommended by the manufacturer. Fire polymerase, Hot-Fire polymerase (Solis Biodyne, Estonia), or the GC-Rich PCR System (Roche, Switzerland) was used in PCR for expression analyses and riboprobe template plasmid construction, all according to the manufacturer’s instructions. An annealing temperature of 57°C was used for all combinations of primers. Cycle numbers were optimized so that the PCR products were analyzed in the exponential phase of the PCR. Depending on the primers used, 30–40 cycles of PCR were performed. PCR fragments were resolved by agarose gel electrophoresis. PCR with primers specific for the ubiquitously expressed glyceraldehyde-3-phosphate dehydrogenase (*GAPDH*), 25 cycles, was performed as a control to determine the amount of human template cDNA in different PCRs. For normalizing the amount of mouse template cDNA and the amount of template cDNA in the panel of human brain regions, a 30-cycle PCR with primers specific for the ubiquitously expressed hypoxanthine guanine phosphoribosyl transferase (*HPRT*) was performed. All PCRs were performed in a volume of 10 μl containing 1/80 of the reverse transcription reaction product as a template. All PCR products were verified by sequencing.

### In situ hybridization

For both human and mouse the in situ hybridization probes were generated as follows. The NFAT riboprobe fragments were selected to cover the parts of the NFAT mRNAs that are present in all mainly expressed splice variants of *NFATC1, NFATC2, NFATC3,* or *NFATC4*. DNA fragments amplified for NFAT riboprobe generation (Supplementary Table 2) were excised from the gel and cloned into pSC-A PCR cloning vector (Stratagene, USA). The mouse NFAT plasmids were linearized adjacent to the 5′ ends of the cloned fragments. The hybridization probes for mouse were 519, 524, 436, and 831 nt in length for *Nfatc1, Nfatc2, Nfatc3,* and *Nfatc4,* respectively. For *Nfatc4* the template for the second assay was generated by digestion at the BglII site inside the cloned *Nfatc4* sequence and the length of the probe was 456 bp. For human in situ hybridization the plasmids with *NFATC1, NFATC2, NFAT3,* and *NFATC4* were linearized with Eco45III, NcoI, NcoI, or HindIII restriction enzyme inside the cloned sequences, respectively. The lengths of the resulting riboprobes were 697 nt for *NFATC1,* 615 nt for *NFATC2,* 876 nt for *NFATC3,* and 816 nt for *NFATC4.* cRNA probes were synthesized with a MAXIScript in vitro transcription kit (Ambion), using [α-^35^S]UTP (Amersham Biosciences) for labeling, according to the manufacturer’s instructions. Serial sagittal and coronal sections (20 μm) from fresh-frozen adult male Black 6 mouse brains and coronal sections (16 μm) from fresh-frozen adult male human hippocampus were subjected to in situ hybridization using a protocol described elsewhere by Timmusk et al. [54]. Emulsion-dipped sections were developed after 7 weeks using D-19 developer (Eastman Kodak, USA), fixed (sodium fixer; Kodak), and counterstained with hematoxylin (Shandon, USA).

## Figures and Tables

**Table 1:** Usage of alternative 5′ and 3′ exons and alternative splicing of human and mouse NFAT genes

Gene			Human	Mouse
*NFATC1*	5′ exons	IA	[24]	[41]
		IB	[29]	[42]
	3′ exons	VIII	[24]	[42]
		X	[29]	[41]
	Alternative splicing	IXL	[45]	+
		IXS	[29]	[41]
		ΔIX	+	ND
*NFATC2*	5′ exons	IA	+	+
		IB	[43]	[44]
	3′ exons	III	ND	+
		VIIA	ND	[33]
		X	[43]	[43]
	Alternative splicing	IIL	[43]	[43]
		IIS	+	+
		ΔII	ND	+
		XA	[43]	[43]
*NFATC3*	5′ exons	IAL	+	[32]
		IB	[23]	[46]
		IC	+	ND
		ID	+	ND
		IE	+	ND
		IF	+	ND
	3′ exons	IV	ND	+
		IX	[26]	ND
		X	[26]	[32]
	Alternative splicing	IAS	+	ND
		XA	[23]	[46]
		XB	[26]	ND
*NFATC4*	5′ exons	IA	+	ND
		IB	+	ND
		IC	+	ND
		ID	[23]	[1]
		IE	+	ND
		IV	+	ND
		VI	+	+
	3′ exon	X	[23]	[1]
	Alternative splicing	IEi	+	ND
		VIi	ND	+
		IXL	[23]	[1]
		IXS	+	ND
		IXi	+	+

+, novel transcript identified in this study; ND, not detected; references indicate studies describing the respective transcript variant.

## Appendix A Supplementary data

Supplementary Table 1 Transcript types of human and mouse *NFAT* genes.

Supplementary Table 2 Primers used to analyse the expression of human and mouse *NFAT* mRNAs:
