# Supplementary material for: Alternative splicing and expression of human and mouse NFAT genes
Source: Genomics. 2008 Nov;92(5):279–91. doi: 10.1016/j.ygeno.2008.06.011 (PMC2577130; doi:10.1016/j.ygeno.2008.06.011)
Supplement: Supplementary Table 1 — Transcript types of human and mouse NFAT genes. [file mmc1.doc]

Supplementary Table 1.

Transcript types of human and mouse *NFAT genes*.

| Transcript | Human | | | Mouse | | |
| --- | --- | --- | --- | --- | --- | --- |
|  | bp | | aa; Mw (Da) | bp | | aa; Mw (Da) |
| *NFATc1-IA-IXL* | 2829 | | 943; 101243 | 2817 | | 939; 101610 |
| *NFATc1-IB-IXL* | 2790 | | 930; 100336 | 2775 | | 925; 100468 |
| *NFATc1-IA-∆IX* | 2139 | | 713; 77376 | ND | | |
| *NFATc1-IB-∆IX* | 2100 | | 700; 76469 | ND | | |
| *NFATc1-IA-IXS* | 2475 | | 825; 88763 | 2481 | 827; 89471 | |
| *NFATc1-IB-IXS* | 2436 | | 812; 87857 | 2439 | 813; 88328 | |
| *NFATc1-IA-VIII* | 2148 | | 716; 77785 | 2151 | 717; 77834 | |
| *NFATc1-IB-VIII* | 2109 | | 703; 76879 | 2109 | 703; 76692 | |
| *NFATc2-IA-IIL-Xa* | 2703 | | 901; 97693 | 2709 | 903; 97617 | |
| *NFATc2-IB-IIL-Xa* | 2763 | | 921; 99784 | 2769 | 923; 99642 | |
| *NFATc2-IA/IB-IIS-Xa* | 2106 | | 702; 76700 | 2106 | 702; 76589 | |
| *NFATc2-IA/IB-∆II-Xa* | ND | | | 1419 | 473; 52311 | |
| *NFATc2-IA-IIL-∆Xa* | 2715 | | 905; 98054 | 2721 | 907; 97995 | |
| *NFATc2-IB-IIL-∆Xa* | 2775 | | 925; 100146 | 2781 | 927; 100020 | |
| *NFATc2-IA/IB-IIS-∆Xa* | 2118 | | 706; 77061 | 2118 | 706; 76966 | |
| *NFATc2-IA/IB-∆II-∆Xa* | ND | | | 1431 | 477; 52689 | |
| *NFATc2-IA-IIL-III* | ND | | | 1377 | 459; 48661 | |
| *NFATc2-IB-IIL-III* | ND | | | 1437 | 479; 50686 | |
| *NFATc2-IA/IB-IIS-III* | ND | | | 774 | 258; 27633 | |
| *NFATc2-IA/IB-∆II-III* | ND | | | 144 | 48; 5423 | |
| *NFATc2-IA-IIL-VIIa* | ND | | | 1959 | 653; 69937 | |
| *NFATc2-IB-IIL-VIIa* | ND | | | 2019 | 673; 71962 | |
| *NFATc2-IA/IB-IIS-VIIa* | ND | | | 1356 | 452; 48908 | |
| *NFATc2-IA/IB-∆II-VIIa* | ND | | | 669 | 223; 24631 | |
| *NFATc3-IAL-Xa* | 1767 | | 589; 63608 | 3180 | 1060; 114491 | |
| *NFATc3-IAL-∆Xa* | 1788 | | 596; 63966 | 3204 | 1068; 114957 | |
| *NFATc3-IAL-IV* | ND | | | 1671 | 557; 60907 | |
| *NFATc3-IB-Xa* | 3204 | | 1068; 115237 | 3204 | 1068; 115071 | |
| *NFATc3-IB-∆Xa* | 3225 | | 1075; 115594 | 3228 | 1076; 115537 | |
| *NFATc3-IB-IX* | 3135 | | 1045; 112642 | ND | | |
| *NFATc3-IB-IV* | ND | | | 1695 | 565; 61486 | |
| *NFATc3-IAS/IC/ID/IE/IF-Xa* | 1767 | 589;63608 | | ND | | |
| *NFATc3-IAS/IC/ID/IE/IF-∆Xa* | 1788 | 596; 63966 | | ND | | |
| *NFATc3-IAL/IAS/IC/ID/IE/IF-IX* | 1698 | 566; 61014 | | ND | | |
| *NFATc4-IA-IXL* | 2895 | 965; 101680 | | ND | | |
| *NFATc4-IA-IXS* | 2571 | 857; 90525 | | ND | | |
| *NFATc4-IA-IXi* | 2892 | 964; 101513 | | ND | | |
| *NFATc4-IB-IXL* | 2745 | 915; 96662 | | ND | | |
| *NFATc4-IB-IXS* | 2421 | 807; 85508 | | ND | | |
| *NFATc4-IB-IXi* | 2742 | 914; 96495 | | ND | | |
| *NFATc4-IC-IXL* | 2802 | 934; 98436 | | ND | | |
| *NFATc4-IC-IXS* | 2478 | 826; 87282 | | ND | | |
| *NFATc4-IC-IXi* | 2799 | 933; 98269 | | ND | | |
| *NFATc4-ID-IXL* | 2706 | 902; 95449 | | 2703 | 901; 95782 | |

| Transcript | Human | | Mouse | |
| --- | --- | --- | --- | --- |
|  | bp | aa; Mw (Da) | bp | aa; Mw (Da) |
| *NFATc4-ID-IXS* | 2382 | 794; 84294 | ND | |
| *NFATc4-ID-IXi* | 2703 | 901; 95282 | 2670 | 890; 94459 |
| *NFATc4-IE-IXL* | 2670 | 890; 94146 | ND | |
| *NFATc4-IE-IXS* | 2346 | 782; 82991 | ND | |
| *NFATc4-IE-IXi* | 2667 | 889; 93978 | ND | |
| *NFATc4-IEi-IXL* | 2496 | 832; 88270 | ND | |
| *NFATc4-IEi-IXS* | 2172 | 724; 77115 | ND | |
| *NFATc4-IEi-IXi* | 2493 | 831; 88103 | ND | |
| *NFATc4-IV-IXL* | 1311 | 437; 47373 | ND | |
| *NFATc4-IV-IXS* | 987 | 329; 36219 | ND | |
| *NFATc4-IV-IXi* | 1308 | 436; 47206 | ND | |
| *NFATc4-VI-IXL* | 570 | 190; 20104 | 630 | 210; 23170 |
| *NFATc4-VI-IXS* | 246 | 82; 8950 | ND | |
| *NFATc4-VI-IXi* | 567 | 189; 19937 | 744 | 248; 27055 |
| *NFATc4-VIi-IXL* | ND | | 630 | 210; 23170 |
| *NFATc4-VIi-IXi* | ND | | 744 | 248; 27055 |
