# Supplementary material for: Alternative splicing and expression of human and mouse NFAT genes
Source: Genomics. 2008 Nov;92(5):279–91. doi: 10.1016/j.ygeno.2008.06.011 (PMC2577130; doi:10.1016/j.ygeno.2008.06.011)
Supplement: Supplementary Table 2 — Primers used to analyse the expression of human and mouse NFAT mRNAs: [file mmc2.doc]

Supplementary Table 2.

1. Primers used to analyse the expression of human and mouse *NFAT* mRNAs:

| Transcript type/  Amplicon size | Primers |
| --- | --- |
| Human *NFATc1* |  |
| *NFATc1-IA*/  1542 bp | hmrNFATc1exIAs, ACCAGCTTTCCAGTCCCTTCCAAG  hmrNFATc1exIVas, GGGATCTCCAGGACTTTGGTGTTG |
| *NFATc1-IB*/  1575 bp | hNFATc1exIBs, TCCTCTTCGAGTTTAACCAGCG  hrNFATc1exIVas, GGGATCTCCAGGACTTTGGTGTTG |
| *NFATc1-VIII*/  276 bp | hrNFATc1exVIIs, TGGCCACCATGTCTGGGAGATG  hNFATc1exVIIIUTRas, TGCTTTACGGCGACGTCGTTTC |
| *NFATc1-∆IX*/  245 bp | hrNFATc1exVIIs, TGGCCACCATGTCTGGGAGATG  hNFATc1exXas, GTGGCAACTAGGAGTGGG |
| *NFATc1-IXS*/  628 bp | hrNFATc1exVIIs, TGGCCACCATGTCTGGGAGATG  hNFATc1exXas, GTGGCAACTAGGAGTGGG |
| *NFATc1-IXL*/  935 bp | hrNFATc1exVIIs, TGGCCACCATGTCTGGGAGATG  hNFATc1exXas, GTGGCAACTAGGAGTGGG |
| Mouse *NFATc1* |  |
| *NFATc1-IA/*  462 bp | hmrNFATc1exIAs, ACCAGCTTTCCAGTCCCTTCCAAG  mNFATc2exIIas, TGCAGGGTTGCTGTAGACGGTG |
| *NFATc1-IB/*  406 bp | mNFATc1exIBs, GGAGTTCGACTTCGATTTCCTC  mNFATc2exIIas, TGCAGGGTTGCTGTAGACGGTG |
| *NFATc1-VIII/*  347 bp | mNFATc1exVIIs, CTGGGAGATGGAAGCAAAGAC  mrNFATc1exVIIIUTRas, GCGACTTGGTCTTGTGAATAGGG |
| *NFATc1-IXS/*  596 bp | mNFATc1exVIIs, CTGGGAGATGGAAGCAAAGAC  mrNFATc1exXas, GTGCTGGAGAGGTCGTTACG |
| *NFATc1-IXL/*  885 bp | mNFATc1exVIIs, CTGGGAGATGGAAGCAAAGAC  mrNFATc1exXas, GTGCTGGAGAGGTCGTTACG |
| Human *NFATc2* |  |
| *NFATc2-IA/*  620 bp | hNFATc2exIAs, CTTTCCAAACACGCGCCAAG  hmNFATc2exII_LONGas, CTGGGGGAATAATGAGCAGGG |
| *NFATc2-IB-IIS/*  154 bp | hNFATc2exIBs, GACTATGAGTATTTGAATCCG  hmrNFATc2exII_BOTHas, GCGCACGAATGCCTCCGCTTG |
| *NFATc2-IB-IIL/*  532 bp | hNFATc2exIBs, GACTATGAGTATTTGAATCCG  hmrNFATc2exII_BOTHas, GCGCACGAATGCCTCCGCTTG |
| *NFATc2-ΔXa/*  857 bp | hmrNFATc2exVIIIs, CATGCTTTTTGTTGAGATCCC  hNFATc2exXas, CTGATTTCTGGCAGGAGGTC |
| *NFATc2-Xa/*  901 bp | hmrNFATc2exVIIIs, CATGCTTTTTGTTGAGATCCC  hNFATc2exXas, CTGATTTCTGGCAGGAGGTC |
| Mouse *NFATc2* |  |
| *NFATc2-IA-IIS/*  245 bp | mNFATc2exIAs, GCCAGATCACAGCACACGGTC  hmNFATc2exII_BOTHas, GCGCACGAATGCCTCCGCTTG |
| *NFATc2-IA-IIL/*  765 bp | mNFATc2exIAs, GCCAGATCACAGCACACGGTC  hmNFATc2exII_BOTHas, GCGCACGAATGCCTCCGCTTG |
| *NFATc2-IB-IIS/*  165 bp | mrNFATc2exIBs, CAAGACGAGCTGGACTTTTC  hmrNFATc2exII_BOTHas, GCGCACGAATGCCTCCGCTTG |
| *NFATc2-IB-IIL/*  703 bp | mrNFATc2exIBs, CAAGACGAGCTGGACTTTTC  hmrNFATc2exII_BOTHas, GCGCACGAATGCCTCCGCTTG |

| Transcript type/  Amplicon size | Primers |
| --- | --- |
| *NFATc2-VIIa/*  635 bp | mrNFATc2exIVs, CACGGCTACATGGAGAACAAG  mrNFATc2exVIIUTRas, GGAAGGAGCACGGAGCATCTGAAG |
| *NFATc2-∆Xa/*  911 bp | hmrNFATc2exVIIIs, CATGCTTTTTGTTGAGATCCC  mNFATc2exXas, GGTCCTGAAAACTCCTTCCTGATG |
| *NFATc2-Xa/*  997 bp | hmrNFATc2exVIIIs, CATGCTTTTTGTTGAGATCCC  mNFATc2exXas, GGTCCTGAAAACTCCTTCCTGATG |
| Human *NFATc3* |  |
| *NFATc3-IAS/*  443 bp | hNFATc3exIAs, CAGCCATTAAAGTTGAGGTGGG  hNFATc3exIIas, CTGCTGGCAGGACTAGGACTA |
| *NFATc3-IAL/*  698 bp | hNFATc3exIAs, CAGCCATTAAAGTTGAGGTGGG  hNFATc3exIIas, CTGCTGGCAGGACTAGGACTA |
| *NFATc3-IB/*  528 bp | hmrNFATc3Ibs, CACGCCGATGACTACTGCAAACTG  hNFATc3exIIas, CTGCTGGCAGGACTAGGACTA |
| *NFATc3-IC/*  470 bp | hNFATc3ICs, GATGACCGGAGACCGATAACCC  hNFATc3exIIas, CTGCTGGCAGGACTAGGACTA |
| *NFATc3-ID/*  494 bp | hNFATc3IDs, CCCTGTTACATATGCATACGAAC  hNFATc3exIIas, CTGCTGGCAGGACTAGGACTA |
| *NFATc3-IE/*  435 bp | hNFATc3exIEs, CCTGAACGTGAGGCATGAGGATTCT  hNFATc3exIIas, CTGCTGGCAGGACTAGGACTA |
| *NFATc3-IF/*  495 bp | hNFATc3exIFs, GGCTGCAGTGGTTTTACATCTCTG  hNFATc3exIIas, CTGCTGGCAGGACTAGGACTA |
| *NFATc3-IX/*  1161 bp | hNFATc3exVIIIs, CCTCCATATCATAACCCAGCAG  hNFATc3exIXUTRas, GTCCCTGAATCTTCACTACTTC |
| *NFATc3-ΔXa/*  91 bp | hNFATc3exIXs, CAGAAGATCGAGAGCCTAACTTTGC  hmNFATc3exXas, CAGAAATCTGGGACATGTCTCTC |
| *NFATc3-Xa/*  195 bp | hNFATc3exIXs, CAGAAGATCGAGAGCCTAACTTTGC  hmNFATc3exXas, CAGAAATCTGGGACATGTCTCTC |
| Mouse *NFATc3* |  |
| *NFATc3-IA/*  190 bp | mNFATc3exIAs, TCTCTGTGTCTGCTCAACTTCCG  mrNFATc2exIIas, CCTTGGAGCTGAAATGATGGTGAC |
| *NFATc3-IB/*  246 bp | hmrNFATc3IBs, CACGCCGATGACTACTGCAAACTG  mrNFATc2exIIas, CCTTGGAGCTGAAATGATGGTGAC |
| *NFATc3-IV/*  392 bp | hmrNFATc3exIIIs, TGAAACTGAAGGTAGCCGAGGG  mNFATc3exIVUTRas, GGCTGGAGATAGAGGCAGGTGA |
| *NFATc3-ΔXa/*  555 bp | mNFATc3exIXs, CGACAGGACATCTCTTAGC  hmNFATc3exXas, CAGAAATCTGGGACATGTCTCTC |
| *NFATx3-Xa/*  659 bp | mNFATc3exIXs, CGACAGGACATCTCTTAGC  hmNFATc3exXas, CAGAAATCTGGGACATGTCTCTC |
| Human *NFATc4* |  |
| *NFATc4-IA/*  1282 bp | hNFATc4exIA, CCGTTTAGTTGCTGGGATGGGGC  hNFATc4exIIas, GGATGCTCTCAGCTGGTGGGGCC |
| *NFATc4-IB/*  1100 bp | hNFATc4exIB, GAAGAGGAGGGGAACCCACAGG  hNFATc4exIIas, GGATGCTCTCAGCTGGTGGGGCC |
| *NFATc4-IC/*  1094 bp | hNFATc4exIC, CTTTGGGGGTCCTGGAGGAATGGC  hNFATc4exIIas, GGATGCTCTCAGCTGGTGGGGCC |

| Transcript type/  Amplicon size | Primers |
| --- | --- |
| *NFATc4-ID/*  689 bp | hNFATc4exIDs, ACCCGGGTGAAGATACAGCAG  hNFATc4exIIas, CTCATTTAGCTCAGACTCCACCTC |
| *NFATc4-IE.*  613 bp | hNFATc4exIeS, GTCCTAGGATCCAGGGGCCAGTG  hNFATc4exIIas, CTCATTTAGCTCAGACTCCACCTC |
| *NFATc4-IEi/*  738 bp | hNFATc4exIeS, GTCCTAGGATCCAGGGGCCAGTG  hNFATc4exIIas, CTCATTTAGCTCAGACTCCACCTC |
| *NFATc4-IV/*  536 bp | hNFTAc4exIVUTRs, GGATGAGACGGTGGGGATTTC  hmrNFATc4exVIas, GAGTCTGGCAGGAAGTTGGA |
| *NFATc4-VI/*  429 bp | hNFATc4exIV5’UTRs, GTTTAACCCTCTCTCTGCTCTG  hmNFATc4exVIIIas, CTTGTTGCTGTACTCGGGGA |
| *NFAT-IXS/*  492 bp | hNFATc4exVIIs, AGGAGGCCACAGTGAACCGA  hmrNFATc4exXas, TTCAGGCAGGAGGCTCTTCTC |
| *NFATc4-IXL/*  816 bp | hNFATc4exVIIs, AGGAGGCCACAGTGAACCGA  hmrNFATc4exXas, TTCAGGCAGGAGGCTCTTCTC |
| *NFATc4-IXi/*  1575 bp | hNFATc4exVIIs, AGGAGGCCACAGTGAACCGA  hmrNFATc4exXas, TTCAGGCAGGAGGCTCTTCTC |
| Mouse *NFATc4* |  |
| *NFATc4-VI/*  277 bp | mrNFATc4exVI5’UTRs, CACTCAGCCCAGCCAGGCTTC  hmNFATc4exVIIIas, CTTGTTGCTGTACTCGGGGA |
| *NFATc4-VIi/*  1332 bp | mrNFATc4exVI5’UTRs, CACTCAGCCCAGCCAGGCTTC  hmNFATc4exVIIIas, CTTGTTGCTGTACTCGGGGA |
| *NFATc4-IXL/*  831 bp | mNFATc4exVIIs, GAAAACTGCAGTGGGAGGAAGAGG  hmrNFATc4exXas, TTCAGGCAGGAGGCTCTTCTC |
| *NFATc4-IXi/*  1583 bp | mNFATc4exVIIs, GAAAACTGCAGTGGGAGGAAGAGG  hmrNFATc4exXas, TTCAGGCAGGAGGCTCTTCTC |
| Human *GAPDH* |  |
| *GAPDH/*  261 bp | GAPDHs, TCCCCACTGCCAACGTGTCAGTG  GAPDHas, ACCCTGTTGCTGTAGCCAAATTCG |
| Mouse *HPRT* |  |
| *HPRT/*  331 bp | hmrHPRTs, GATGATGAACCAGGTTATGAC  hmrHPRTas, GTCCTTTTCACCAGCAAGCTTG |

1. Primers used for mouse *NFAT* riboprobe generation:

| Mouse gene  Amplicon size | Primers |
| --- | --- |
| *NFATc1/*  519 bp | insitu_mNFATc1exIVs, GCTACAGCTGTTCATTGGG  insitu_mNFATc1exVIIas, GTCTTTGCTTCCATCTCCCAG |
| *NFATc2/*  524 bp | insitu_mNFATc2exIIIs, CGCTGTCAAAGCCCCAACAGGA  insitu_mNFATc2exVIas, CCTTGGACTCCGCTGTGAAG |
| *NFATc3/*  436 bp | insitu_mNFATc3exIIs, CCCTGCACCGTTTCCATTTCAGTAC  insitu_mNFATc3exIIIas, CTTCACAACAGGATGGCCACCAGT |
| *NFATc4/*  831 bp | mNFATc4exVIIs, GAAAACTGCAGTGGGAGGAAGAGG  hmrNFATc4exXas, TTCAGGCAGGAGGCTCTTCTC |

1. Primers used for human *NFAT* riboprobe generation:

| Human gene  Amplicon size | Primers |
| --- | --- |
| *NFATc1/*  2479 bp | hNFATc1ex1A_ATGs, CACCATGCCAAGCACCAGCTTTCC  hNFATc1ex9S_woSTOPas, CTGCTGTGGCAGCAGGGCCGG |
| *NFATc2/*  2715 bp | hNFATc2ex1A_ATGs, CACCATGCAGAGAGAGGCTGCGTTCAG  hNFATc2ex10_wSTOPas, GGCTTCTTTTACGTCTGATTTCTGGC |
| *NFATc3/*  1698 bp | hNFATc3ex4_ATGs, CACCATGTTTATTGGGACAGCAGATGATCG  hNFATc3ex9_wSTOPas, GTCCCTGAATCTTCACTACTTC |
| *NFATc4/*  1311 bp | hNFATc4ex4_ATGs, CACCATGTTCATCGGCACTGCAG  hNFATc4ex10_wSTOPas, CAGTTCACGTGGTTCAGGCAGGAGG |
